# Prenatal screening of fetal ventriculoarterial connections: benefits of 4D technique in fetal heart imaging

**DOI:** 10.1186/s12947-017-0108-5

**Published:** 2017-06-23

**Authors:** Yu Wang, Miao Fan, Faiza Amber Siddiqui, Meilian Wang, Wei Sun, Xue Sun, Wenjia Lei, Ying Zhang

**Affiliations:** 1 Department of Sonography, Shengjing Hospital of China Medical University, Heping District, Shenyang, China; 2 Department of Radiology, The first Affiliated Hospital of Sun Yat-sen University, Guangzhou, China; 3 Department of Entomology, The Pennsylvania State University, University Park, PA 16802 USA; 4 Department of Microbiology and Parasitology, College of Basic Medical Sciences, China Medical University, Heping District, Shenyang, China

**Keywords:** Fetal echocardiography, Conotruncal anomalies, 4D, Spatiotemporal image correlation, STIC

## Abstract

**Background:**

Identification of prenatal ventriculoarterial connections in fetuses with conotruncal anomalies (CTA) remains one of the greatest challenges for sonographers performing screening examinations. Herein, we propose a novel protocol of 4D volume analysis that identifies ventriculoarterial connections and evaluate its clinical utility in routine screenings.

**Methods:**

Twenty-nine cases of transposition of the great arteries (TGA), 22 cases of double-outlet right ventricle (DORV), 36 cases of tetralogy of Fallot (TOF), 14 cases of truncus arteriosus (TCA), and randomly selected 70 normal fetuses were reviewed in this study. All cases were evaluated using 2D data alone (2D method), post-processing volumes with no exact algorithm (4D-1 method), or with the proposed algorithm (4D-2 method), or using the 2D and 4D data together (combined method). Comparisons were made to evaluate the detection rate of ventriculoarterial connections for these different methods.

**Results:**

During 18–28 gestational weeks, the detection rate of 4D-2 modality was satisfactory. The detection rate of the combined method was significantly higher than 2D method in the identification of TGA, TOF, and TCA. The detection rate of 4D-1 method was significantly lower than 4D −2 modality for CTA fetuses. During late pregnancy, the detection rate for both 4D modalities was very low due to the poor quality of the 4D volumes.

**Conclusions:**

We proposed a detailed protocol, which allowed the examiner to identify fetal ventriculoarterial connections by 4D volumes. Inclusion of blood information into the volumes improved diagnosis. Our findings suggest that the incorporation of 4D STIC into routine screenings could improve the detection for TGA, TOF, and TCA.

**Electronic supplementary material:**

The online version of this article (doi:10.1186/s12947-017-0108-5) contains supplementary material, which is available to authorized users.

## Background

Congenital heart disease (CHD), accounting for about 2.4 to 13.7 per 1000 live births [1], is the most common congenital malformation leading to perinatal morbidity and mortality and is considered the leading cause of death in newborn with congenital anomalies [2, 3]. Of all CHDs, up to 10–30% are conotruncal anomalies (CTA), which are characterized by a defect in the conotruncal septum arising from an abnormal cardiac neural crest cell migration [4–7]. As a result of errors during embryogenesis, a spectrum of various malformations could result, among which the most common include tetralogy of Fallot (TOF), transposition of the great arteries (TGA), double-outlet right ventricle (DORV) and truncus arteriosus (TCA).

Fetal two-dimensional echocardiography (2DE) and color Doppler echocardiography (CDE) are currently the primary techniques for the diagnosis of fetal CHD. During the past 20 years, the prenatal diagnostic ability of CHD has been promoted dramatically due to the high resolution 2D ultrasound and the advances in Doppler technology [8]. But as far as some complex CTA are concerned, the examiner needs to perform a thorough fetal echo to visualize the outflow tracts and the great arteries in detail, which often goes beyond the capability of sonographers performing routine prenatal ultrasound [9–11].

The introduction of 4D ultrasound provides an additional method to evaluate fetal cardiac structures [12]. New modalities for motion-gated cardiac scanning and application of spatiotemporal image correlation (STIC) technique allow the post-processing of the 4D volume datasets and subsequently evaluate cardiac anatomy with the use of multi-planar slicing or surface rendering of 4D volumes [13–16]. This single 4D volume technique allows the identification of different cardiac planes and often times, cardiac structures that are not demonstrated by 2DE techniques [17]. Various reports [18, 19] have demonstrated the value of 4D STIC in the diagnosis of fetal CHDs; however, its application has been limited mainly because of the difficulty in the post analysis of 4D volumes.

The primary objective of this report was to propose a novel protocol of 4D volume analysis in identification of fetal ventriculoarterial connections. We also investigated whether the 4D modality could help in improving the detection for fetal CTA in routine screenings.

## Methods

### Population study

This was a retrospective study. Fetal echocardiograms at Shengjing Hospital of China Medical University from Jan 2010 to Dec 2016 were reviewed to search the cases of DORV, TGA, TOF, and TCA. All TGA fetuses included in the study complicated with ventricular septal defect (VSD). Fetuses with complex CHDs were excluded from the current study. Cases without confirmation were also excluded. In total, 29 cases of TGA, 22 cases of DORV, 36 cases of TOF, and 14 cases of TCA were included in the study. For DORV fetuses, parallel great arteries (aorta on the left/left-anterior/right-anterior to the pulmonary artery) were found in 18 cases, while orthogonal great arteries were demonstrated in the other 4 cases. Gestational age ranged from 18 to 36 (median, 27.8) weeks. All cases were confirmed by the result of follow-up, including postnatal echocardiography, computed tomographic angiography, and the postmortem findings. Randomly selected 70 fetuses with normal cardiac structures were also included in the study. Intracardiac anatomy of all fetuses were confirmed by postnatal echocardiogram. Gestational age ranged from 18 to 36 (median, 26.4) weeks. All the normal and CHD fetuses were singletons. As the gestational age might affect the detection of fetal cardiac structures for 4D modality, the fetuses were divided into two groups according to gestational age (gestational weeks, GW: 18–28 and 29–36). Fetal traditional echocardiography (including 2DE and CDE) and 4D volume acquisition were performed in all the fetuses and the data were saved as video clips and 4D volumes.

### Ultrasonography technique

The patients were examined using either a 4D ultrasound system (Voluson 730 Expert, GE Healthcare, Kretztechnik, Zipf, Austria) or a 4D ultrasound system (Voluson E8, GE Healthcare, Kretztechnik, Zipf, Austria). They were equipped with a 4-8 MHz transabdominal transducer and STIC respectively. Traditional 2DE and CDE was performed on all fetuses and four chamber view (4CV), left and right outflow tract view, and the three-vessel and trachea view were acquired and stored as digital video clips. STIC volume acquisition was performed when a clear 4CV was identified with apical or lateral insonation of the fetal heart. STIC volumes were acquired using both gray-scale and color. If the condition was not satisfied, the patient was asked to walk for 30 min and then return to the examination. Usually, the echo window for the scanning is suitable for STIC acquisition. The woman was asked to hold her breath during each of three STIC volume acquisitions, which were performed with an automatic sweep using the motorized curved-array transducer. The acquisition time ranged from 10 to 15 s, and the sweep angle ranged from 25 to 40°. This increased with gestational age. The volume datasets included the upper fetal mediastinum and the gastric vacuole. The STIC volumes were reconstructed immediately and displayed in a cine loop and then stored for later offline analysis with the PC software (4D Viewer, version 14.0; GE Medical Systems, Zipf, Austria).

### Volume quality classification

Each volume was evaluated and classified on a scale of 1 to 5 adapted from Goncalves et al. [20], according to image quality (1, unacceptable; 2, marginal; 3, acceptable; 4, good; 5, excellent). The main determinants of image quality were the fetal motion artifact, shadowing artifact, and the sharpness of the image.

### 4D volume datasets analysis to identify ventriculoarterial connections

STIC volume datasets analysis was made to access the originating, coursing and spatial relationship of the two great arteries. All volume datasets were displayed using the multi-planar modality. Three orthogonal planes (Panel A represented the transverse view, Panel B represented the sagittal view, and Panel C represented the coronal view) were displayed simultaneously. We then used two protocols in evaluating ventriculoarterial connections by post-processing the 4D volumes.

#### Protocol A (4D-1 modality): Post-processing 4D volumes of gray-scale only

In this protocol, the sonographer was not required to use specific steps to post-process the 4D volumes. Instead, they could adjust the slice position (moving forward or backward) in Panel A, B, and C, to show cardiac structures they determined to be interesting, or they could rotate the images in the x-, y-, and z-axes in the three Panels to obtain the views which could help the diagnosis.

#### Protocol B (4D-2 modality): Post-processing 4D volumes of both gray-scale and blood flow.

**Fig. 1:**
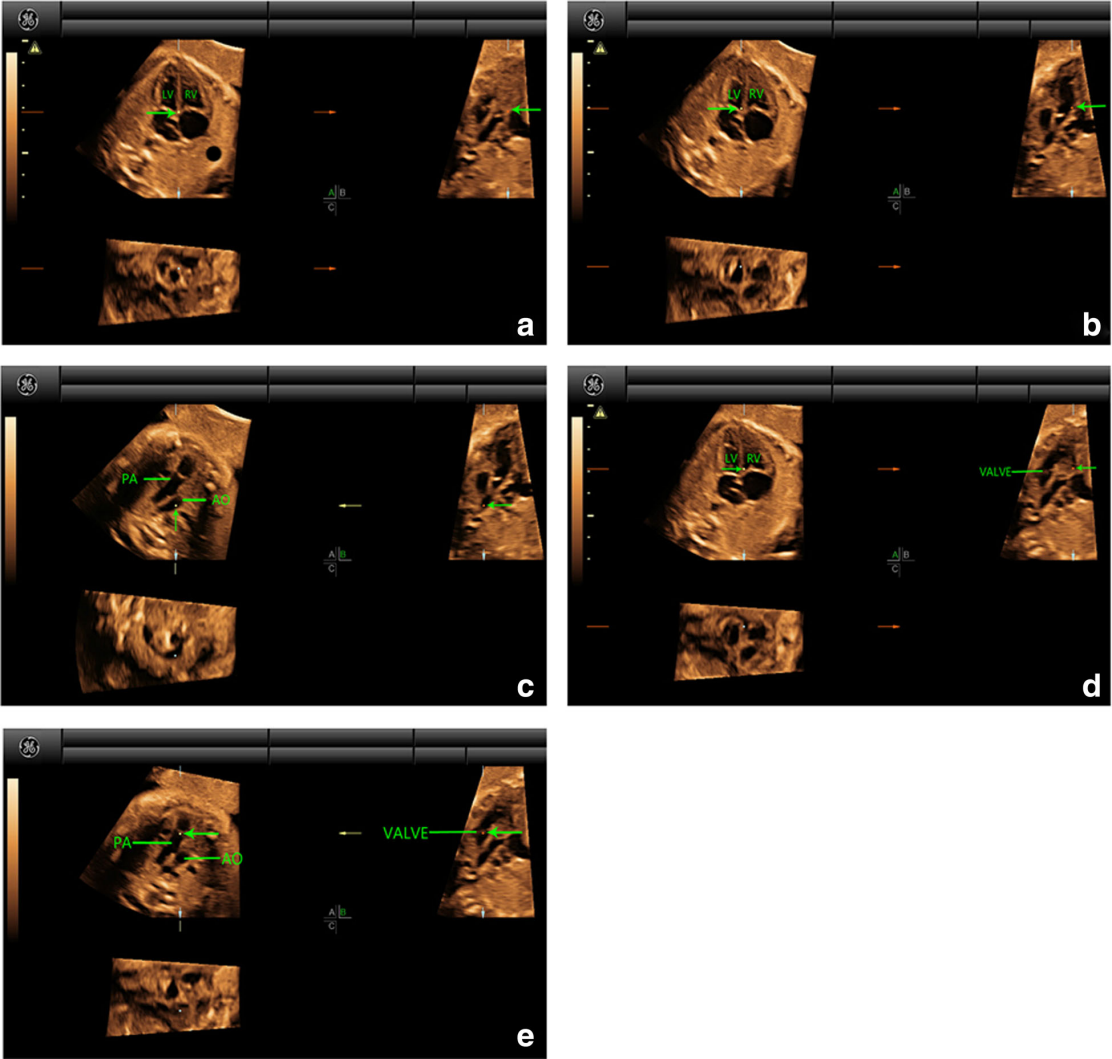
Multiplanar slicing of a fetus with normal cardiac structure of 19 gestational weeks. Panels **a**, **b**, and **c** represent three orthogonal planes (A, transverse; B, sagittal; and C, coronal). The cardiac apex in the 4CV was oriented upwards and the reference point (indicated by the *green* arrow) was placed in the crux of the heart in Panel **a** (*A*). Move the reference point to the left ventricle in Panel **a**. By adjusting the position of the reference point in Panel **a**, near the position of the outflow tract (at the basal part of the left ventricle, near the crux of the heart), a great artery with clear course could be visualized in Panel **b** (*B*). Moving the reference point to this artery and then navigating along the course of the artery in Panel B, we could demonstrate a round (transverse) cross section of one great artery and a longitudinal section of the other great artery (characterized by the short trunk with bifurcation) (*C*). They were aorta and the pulmonary artery. As the reference point was located at the aorta, it could be confirmed that aorta was originated from the left ventricle. Back to the initial state in Panel **a** (*A*). Move the reference point to the right ventricle in Panel **a**. By adjusting the position of the reference point in Panel A, near the position of the outflow tract (at the basal part of the right ventricle, near the crux of the heart), a great artery with its valve could be clearly visualized in Panel **b** (*D*). Moving the reference point to the valve in Panel **b**, two great arteries with cross section and longitudinal section respectively could be visualized in Panel **a** (*E*). As the reference point was located at the pulmonary artery, it could be confirmed that the pulmonary artery was originated from the right ventricle. AO, aorta; LV, left ventricle; PA, pulmonary artery; RV, right ventricle

**Fig. 2:**
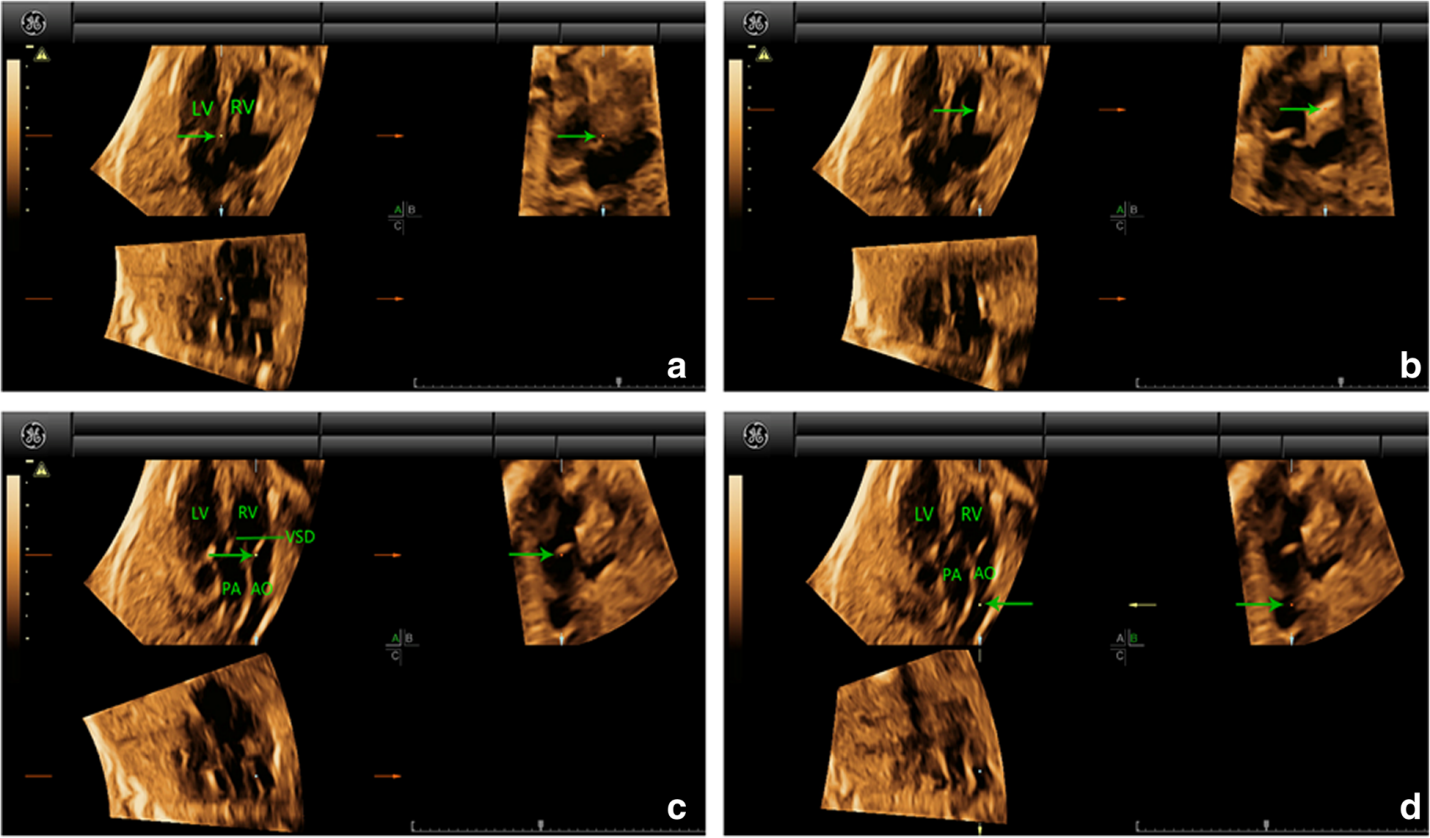
Multiplanar slicing of TGA in a fetus of 25 gestational weeks. Panels **a**, **b**, and **c** represent three orthogonal planes (A, transverse; B, sagittal; and C, coronal). The cardiac apex in the 4CV was oriented upwards and the reference point (indicated by the *green* arrow) was placed in the crux of the heart in Panel **a** (*A*). Move the reference point to the right ventricle in Panel **a**. By adjusting the position of the reference point in Panel **a**, a great artery connecting to the right ventricle could be visualized in Panel **b** (*B*). Moving the reference point to the valve (*C*) and then navigating along the artery (*D*) in Panel **b**, we could then confirm that aorta was originated from the right ventricle and the pulmonary artery (characterized by the short trunk with bifurcation) from the left in Panel **a**. The parallel relationship of the two great arteries was also demonstrated. In addition, a VSD in large size could also be identified. AO, aorta; LV, left ventricle; PA, pulmonary artery; RV, right ventricle; TGA, transposition of the great arteries; VSD, ventricular septal defect

**Fig. 3:**
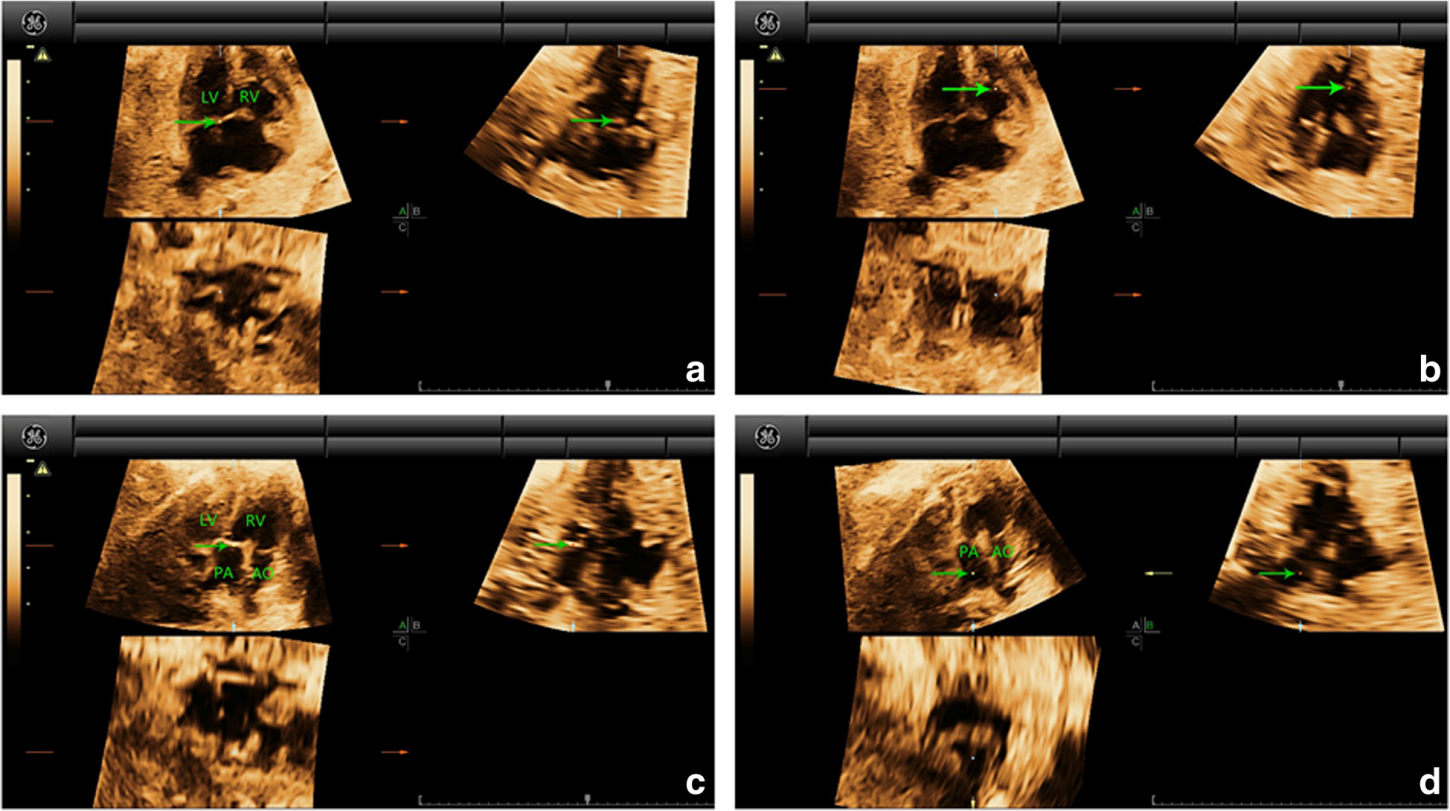
Multiplanar slicing of DORV in a fetus of 26 gestational weeks. Panels **a**, **b**, and **c** represent three orthogonal planes (A, transverse; B, sagittal; and C, coronal). The cardiac apex in the 4CV was oriented upwards and the reference point (indicated by the *green* arrow) was placed in the crux of the heart in Panel **a** (*A*). Move the reference point to the right ventricle in Panel **a**. By adjusting the position of the reference point in Panel *a*, a great artery connecting to the right ventricle could be visualized in Panel **b** (*B*). Moving the reference point to the valve (*C*) and then navigating along the artery (*D*) in Panel **b**, we could then confirm that aorta and the pulmonary artery (characterized by the short trunk with bifurcation) were both originated from the right ventricle and were coursing in parallel in Panel **a**. AO, aorta; DORV, double-outlet right ventricle; LV, left ventricle; PA, pulmonary artery; RV, right ventricle

**Fig. 4:**
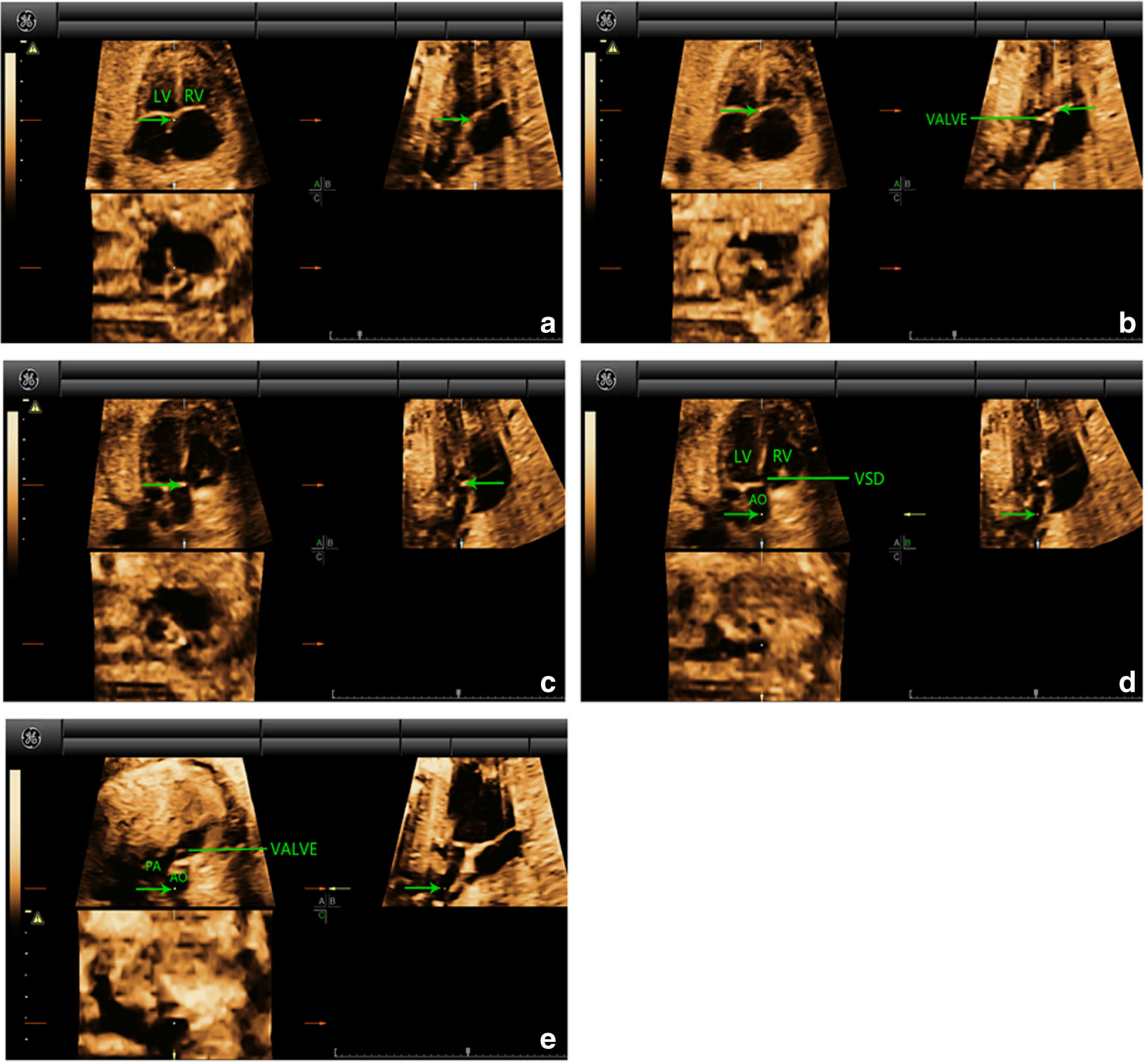
Multiplanar slicing of TOF in a fetus of 24 gestational weeks. Panels **a**, **b**, and **c** represent three orthogonal planes (A, transverse; B, sagittal; and C, coronal). The cardiac apex in the 4CV was oriented upwards and the reference point (indicated by the *green* arrow) was placed in the crux of the heart in Panel **a** (*A*). Move the reference point to the position of the outflow tracts (at the basal part of the ventricles, near the crux of the heart) in Panel **a**. By adjusting the position of the reference point in Panel **a**, a great artery could be visualized in Panel **b** (*B*). Moving the reference point to the valve (*C*) and then navigating along the artery (*D*) in Panel **b**, we could then confirm that aorta was originated from both left and right ventricles in Panel **a**. A large-sized VSD could also be identified. Navigating the reference point further along aorta (*E*) in Panel **b**, we could visualize that the pulmonary artery (characterized by the short trunk with bifurcation) was originated from the right ventricle with a thickened valve in Panel **a**. AO, aorta; LV, left ventricle; PA, pulmonary artery; RV, right ventricle; TOF, tetralogy of Fallot; VSD, ventricular septal defect

**Fig. 5:**
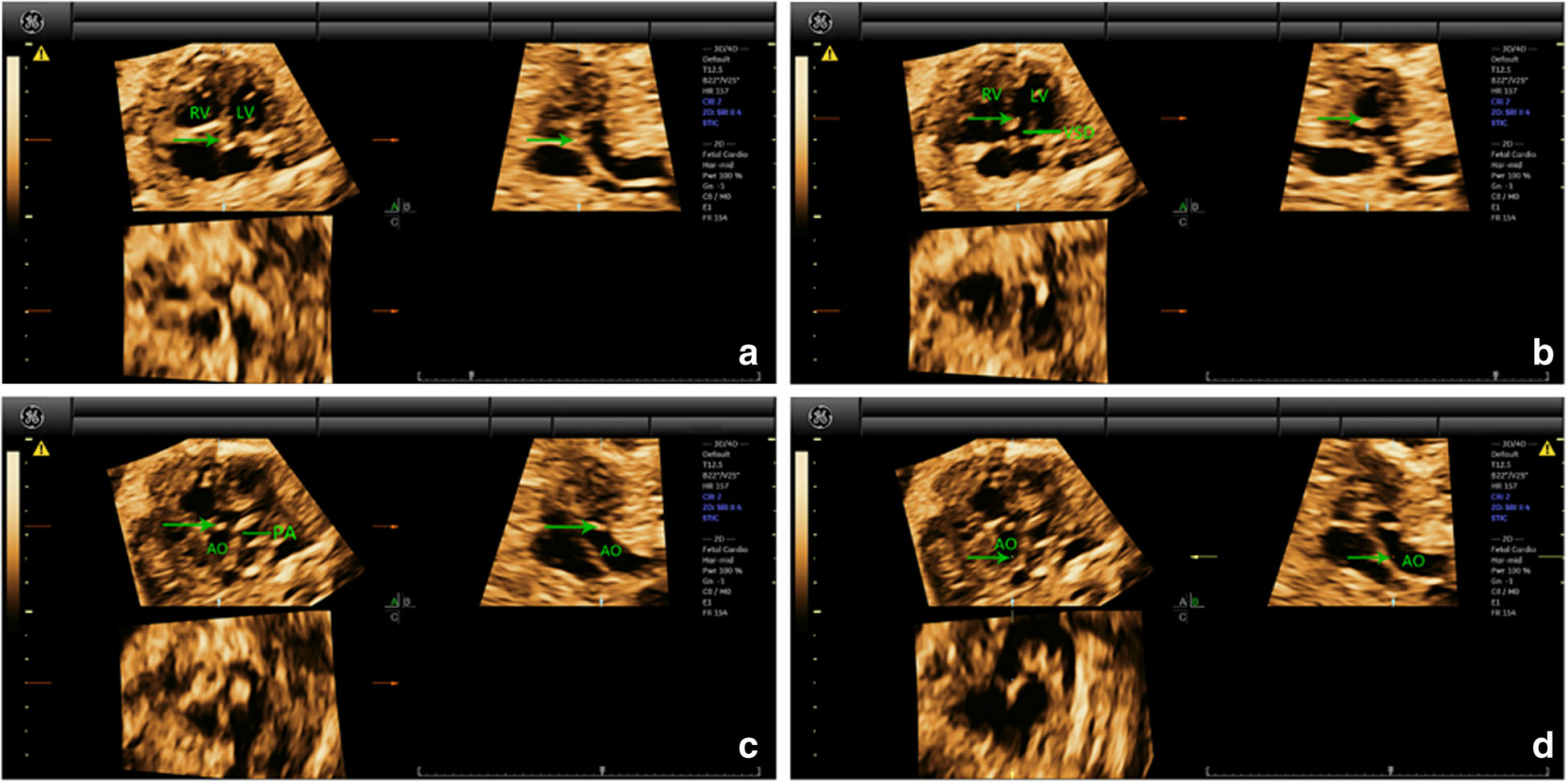
Multiplanar slicing of TCA in a fetus of 28 gestational weeks. Panels **a**, **b**, and **c** represent three orthogonal planes (A, transverse; B, sagittal; and C, coronal). The cardiac apex in the 4CV was oriented upwards and the reference point (indicated by the *green* arrow) was placed in the crux of the heart in Panel **a** (*A*). Move the reference point to the position of the outflow tracts (at the basal part of the ventricles, near the crux of the heart) in Panel **a**. By adjusting the position of the reference point in Panel **a**, a great artery could be visualized in Panel **b**. A VSD could also be identified (*B*). Moving the reference point to the valve (*C*) and then navigating along the artery (*D*) in Panel **b**, we could then confirm that only one great artery arising from both ventricles (mainly from the right ventricle) in Panel **a**. This great artery is aorta. The pulmonary artery was visualized arising from the root of aorta in Panel **a**. AO, aorta; LV, left ventricle; PA, pulmonary artery; RV, right ventricle; TCA, truncus arteriosus; VSD, ventricular septal defect

For the STIC volumes with gray-scale, the cardiac apex in the 4CV was oriented upwards and then the reference point was placed in the crux of the heart in Panel A. The reference point to the ventricles was moved (at the lower part of the ventricle, near the position of the outflow tracts). Navigating the reference point slowly from left to right in Panel A and one or two arteries could be visualized in Panel B. Moving the reference point to the valve and then navigating along the artery in Panel B, we could identify whether aorta or the pulmonary artery originated from one or two ventricles in Panel A. The originating and spatial relationships of the two great arteries were then identified. The processes of navigation of the reference point were described in detail in figures and video clips in a case of normal fetus (Fig. 1, Additional file 1: Movie S1 and Additional file 2: Movie S2), TGA (Fig. 2, Additional file 3: Movie S3), DORV (Fig. 3, Additional file 4: Movie S4 and Additional file 5: Movie S5), TOF (Fig. 4, Additional file 6: Movie S6), and TCA (Fig. 5, Additional file 7: Movie S7), respectively. The time for 4D volume post analysis was recorded. The number of corrected evaluation of ventriculoarterial connections was recorded.

For the STIC volumes with blood flow information, adjusting the images in Panel A was done to show the outlet of the ventricles, and the rendered 4D images showing the great arteries were then displayed in Panel D. Reducing the size of ROI in Panel B facilitated the images in Panel D. A combination of smooth surface and gradient light algorithms and post-processing adjustments were used to improve the image quality.

2D video clips interpretation and 4D volume data analysis (4D-1 modality and 4D-2 modality) was carried out by sonographers A, B, and C, respectively. The sonographers were blinded to the patients’ information, prior sonographic imaging reports, and the findings of the follow-up visit. All the 2D and 4D image data were randomly ordered to analyze. The 2D video clips and the 4D data were then prepared in pairs for each patient. Sonographer C assessed ventriculoarterial connections and made the diagnosis using both the 2D and the 4D data (the combined method). Inter-observer agreement of the 4D methods (4D-1 and 4D-2) for diagnostic accuracy of ventriculoarterial connections was determined by two other sonographers (sonographer A, and D) who reanalyzed 4D volumes, respectively, in all the fetuses. Intra-observer variability of 4D-1 and 4D-2 was determined by reanalysis of 4D volumes by sonographer B and sonographer C, respectively, in all fetuses 30 days later. All the sonographers were screening sonographers with 1-year clinical experience. Furthermore, sonographer C and D were trained for 2 weeks in the proposed protocol (4D-2 modality) for 4D volume post-processing analysis.

### Statistical analysis

The detection rate of ventriculoarterial connections in fetal TGA, DORV, TCA, and TOF was determined as the number of cases in whom 2D or 4D method correctly identified ventriculoarterial connections, expressed as a percentage of the total number of cases of TGA, DORV, TCA, and TOF, respectively. The detection rate of ventriculoarterial connections for different methods were compared via McNemar analysis. The volume quality scores of different gestational periods were compared via independent t-test. *P*-values < 0.05 were considered statistically significant. All statistical analyses were performed using commercially available software (SPSS, release 17.0).

## Results

In the current study, we used different methods to identify fetal ventriculoarterial connections for both CTA fetuses and the normal fetuses. For normal fetuses, ventriculoarterial connections could be identified by traditional 2D method in all fetuses during the full gestation period (18–36 GW). For 4D-2 modality, the detection rate was 100% during the second trimester and decreased dramatically (11 of 20; 55%) during late pregnancy. The detection rate for 4D-1 modality was apparently lower as it varied from 82% (41 of 50) to 35% (7 of 20) for the second and third trimester, respectively. During the third trimester, acoustic shadows often appeared when navigating the reference point in the volumes, which led to invisible of origination of the great arteries. These cases were defined as “unclear” or “uncertain” of ventriculoarterial connections.

The mean time for volume post analysis was 8.4 ± 3.3 min and 9.2 ± 4.1 min for 4D-2 and 4D-1 modality, respectively. The inter-observer and intra-observer variability was 3.3 and 5% for 4D-2, respectively. For 4D-1 modality, the inter-observer and intra-observer variability was 15.7 and 13.3%, respectively.

In total, 101 fetuses with TGA, DORV, TOF, and TCA fetuses were included in the current study. The outcomes of CTA fetuses were summarized in Table 1. We then made comparisons of the different methods in evaluation of ventriculoarterial connections (Table 2).

**Table 1:** Outcomes of the 101 fetuses with conotruncal cardiac anomalies in our study

CHD	*n*	TOP		IUFD		NND		NNA	
		*n*	%	*n*	%	*n*	%	*n*	%
TGA	29	6	20.7%	1	3.4%	2	6.9%	20	70%
DORV	22	8	36.4%	2	9.1%	3	13.6%	9	40.9%
TOF	36	3	8.3%	0	-	2	5.6%	31	86.1%
TCA	14	6	42.9%	1	7.1%	1	7.1%	6	42.9%
Total	101	23	22.8%	4	4%	8	7.9%	66	65.3%

All fetuses of TOP and IUFD were confirmed by autopsy findings. All NND and NNA were confirmed by postnatal echocardiography. Computed tomographic angiography was performed in parts of neonates

*CHD* congenital heart disease, *DORV* double-outlet right ventricle, *IUFD* intrauterine fetal death, *NNA* neonatal alive, *NND* neonatal death, *TCA* truncus arteriosus, *TGA* transposition of the great arteries, *TOF* tetralogy of Fallot, *TOP* termination of pregnancy

**Table 2:** The detection rate of ventriculoarterial connections by different methods

CHD (n)	2D (*n*)	4D-1 (*n*)	4D-2 (*n*)	Combined (*n*)
18–28 GW				
TGA (20)	70% (14)	50% (10)	100%^#^ (20)	100%^#^ (20)
DORV (15)	80% (12)	53.3% (8)	80% (12)	80% (12)
TOF (26)	76.9% (20)	57.7% (15)	80.8% (21)	96.2%^*^ (25)
TCA (10)	60% (6)	30% (3)	80% (8)	100%^*^ (10)
29–36 GW				
TGA (9)	66.7% (6)	22.2% (2)	55.6% (5)	66.7% (6)
DORV (7)	85.7% (6)	28.6% (2)	42.9% (3)	85.7% (6)
TOF (10)	80% (8)	20% (2)	50% (5)	80% (8)
TCA (4)	75% (3)	0	25% (1)	75% (3)

CHD, congenital heart disease; Combined, sonographers made the diagnosis using both the 2D and 4D-2 data; *DORV*, double-outlet right ventricle; *GW*, gestational weeks; *TCA*, truncus arteriosus; *TGA*, transposition of the great arteries; *TOF*, tetralogy of Fallot; Compared with 2D method

^*^ *P* < 0.05; ^#^ *P* < 0.01

**Fig. 6:**
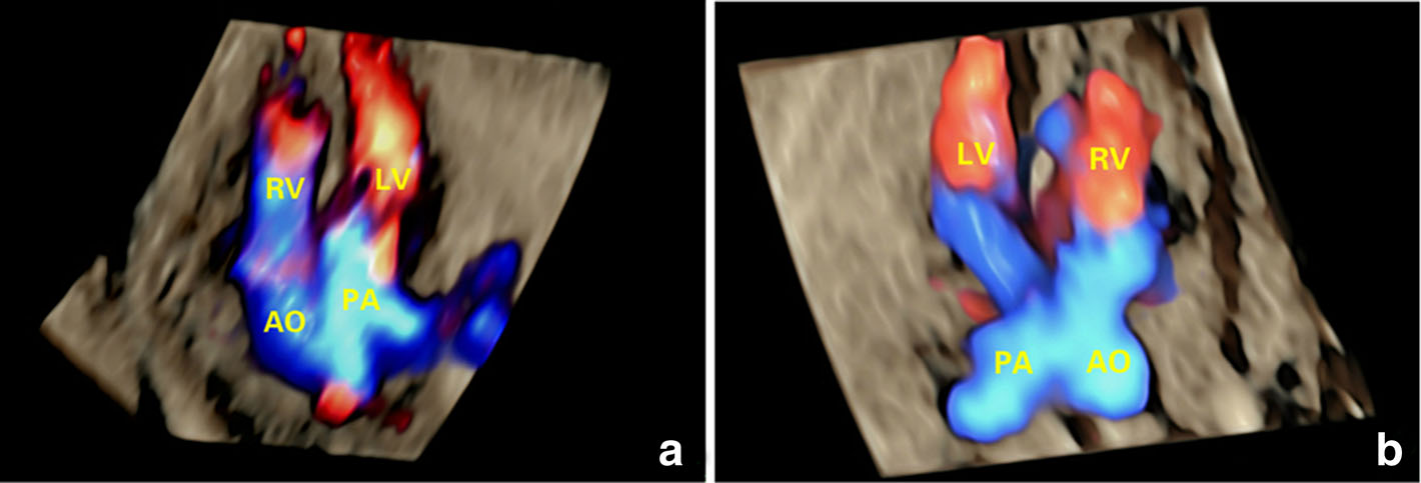
Detection of great arteries alignment in TGA and normal fetuses using 4D volumes with color flow information. In a 24-gestational-week fetus with TGA (**a**), parallel great arteries were clearly identified by the 4D rendered image. The pulmonary artery was originated from the left ventricle while the right ventricle gave off the aorta. In a normal fetus (**b**) of similar gestational age, the 4D rendered image demonstrated that the two great arteries were orthogonal to each other originating from each ventricle. AO, aorta; LV, left ventricle; PA, pulmonary artery; RV, right ventricle; TGA, transposition of the great arteries

During the second trimester, the results showed that both the 2D and 4D-2 modalities provided a satisfactory detection rate for all CTA fetuses. For TGA fetuses, 6 cases were diagnosed as VSD without identification of the discordant of ventriculoarterial connections by 2D method. By navigating the reference point when processing the volumes, the sonographer could clearly visualize the full length of the outflow tracts in all cases. In addition, the 4D–rendered images of the great arteries disclosed parallel-like great arteries (Fig. 6a) which were obviously different from the normal arrangement (Fig. 6b). Statistical analysis showed a significantly higher detection rate for the 4D-2 modality when compared with traditional 2D method.

For DORV fetuses, 12 cases were successfully identified by the traditional 2D method, in which aorta was on the left or left-anterior to the pulmonary artery and the two great arteries lay in parallel. The other 3 cases were misdiagnosed as TOF because the two great arteries were wrapped in relationship. The detection rate of the 4D-2 method was similar to the 2D modality, as the arterial conical connections were not discerned by screening sonographers by either 2D or the 4D modality.

**Fig. 7:**
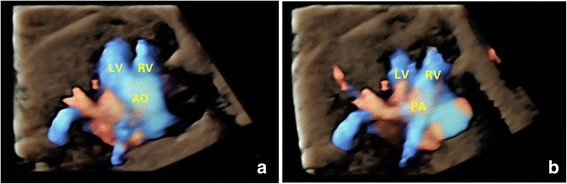
Detection of TOF in a fetus of 24 gestational weeks using 4D volumes with color flow information. The 4D rendered image clearly showed that the large in sized aorta was connected to both ventricles (**a**). Another image showed the stenosis pulmonary artery was connected to the right ventricle (**b**). AO, aorta; LV, left ventricle; PA, pulmonary artery; RV, right ventricle; TOF, tetralogy of Fallot

VSD was identified in 6 of 26 cases of TOF, while the overriding of aorta was not identified by the 2D modality. In 1 of the missed 6 cases, 4D-2 modality made the correct diagnosis as the overriding of aorta could be identified when navigating the reference point in the volumes. At the same time, the origination of aorta from both ventricles and the right ventricle gave off the pulmonary artery, which was clearly visualized by the 4D–rendered image (Fig. 7). It was notable that most cases were identified using the combined method, when the sonographers processing the volumes after reviewing the 2D images (the combined method). Statistical analysis showed a significantly higher detection rate for the combined method than traditional 2D modality.

**Fig. 8:**
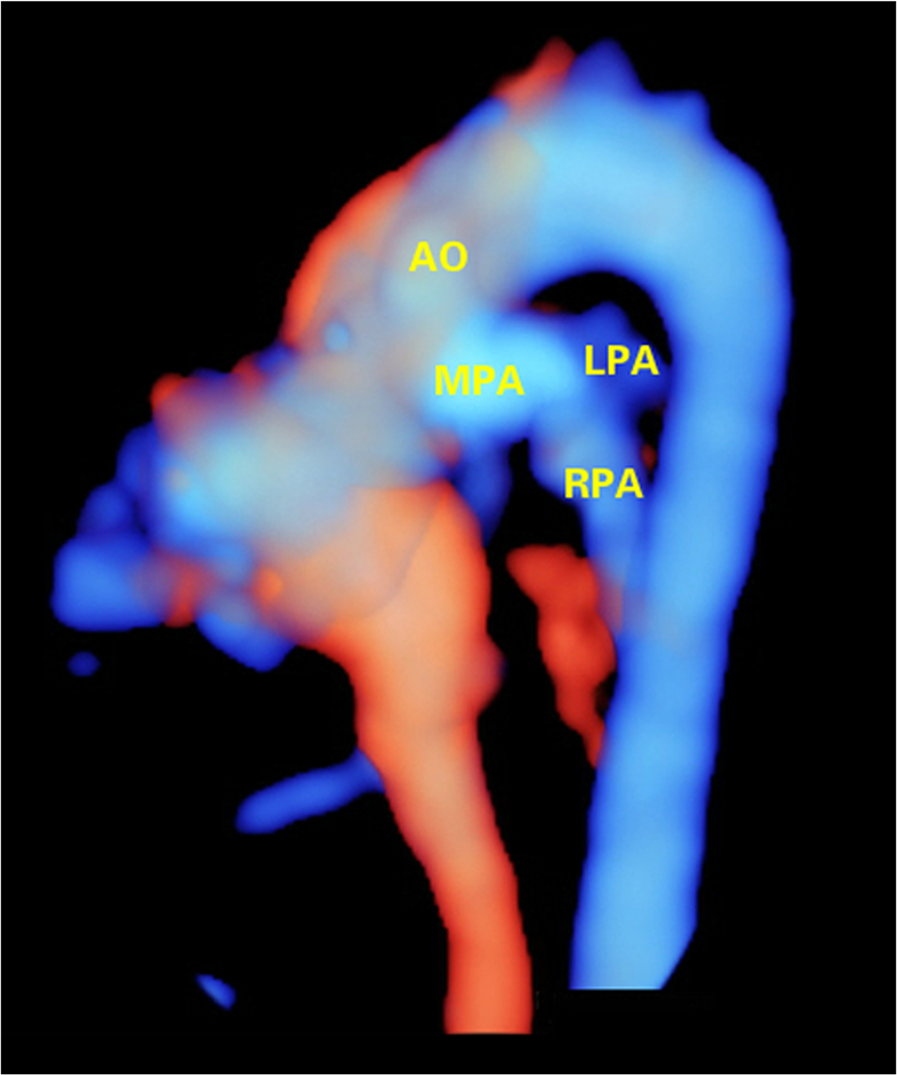
Detection of TCA in a fetus of 28 gestational weeks using 4D volumes with color flow information. The 4D rendered image clearly showed that a large in sized great artery(aorta) was originated from both ventricles. The main pulmonary artery was connected to the root of aorta. AO, aorta; LPA, left pulmonary artery; MPA, main pulmonary artery; RPA, right pulmonary artery; TCA, truncus arteriosus

In 4 of 10 TCA fetuses, the final diagnosis was not reached by the 2D modality, as the origination of the pulmonary artery could not be identified. 4D-2 modality performed better as the 4D rendered images clearly delineated the origination of the pulmonary arteries (Fig. 8). It was interesting that the detection rate was significantly improved compared with traditional 2D method, when the sonographers processed the volumes after reading the 2D images (the combined method).For 4D-1 modality, the detection rate of ventriculoarterial connections was unsatisfactory, as it varied from 30 to 57.7%. The detection rate of ventriculoarterial connections was significantly lower for 4D-1 modality than 4D-2 method.

During the late gestation period, the detection rates of ventriculoarterial connections for both 4D modalities decreased significantly and were lower than 2D modality. The combination of 4D data with 2D data did not improve diagnosis.

The mean time for volume post-analysis was 12.5 ± 4.7 min and 20.5 ± 9.1 min for 4D-2 and 4D-1, respectively. For 4D-2 modality, the sonographer was required to post-process volumes for both gray-scale and color. However, the time needed for post-processing 4D-2 modality was apparently shorter than 4D-1 modality in the detection of CTA fetuses, which confirmed that the proposed protocol could be easily adapted by screening sonographers. The inter-observer and intra-observer variability was 8.2 and 6.8% for 4D-2 modality, respectively. For 4D-1 modality, the inter-observer and intra-observer variability was 18.2 and 15.4%, respectively. The results suggested that 4D-2 modality showed better reproducibility than 4D-1.

**Fig. 9:**
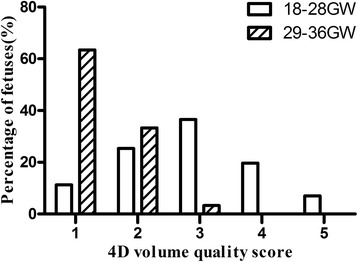
Distribution of STIC volume quality for 101 CTA fetuses. 1, unacceptable; 2, marginal; 3, acceptable; 4, good; 5, excellent; GW, gestational weeks

For the 4D-2 modality, we evaluated the relationship of quality of the acquired volumes and the detection rate of ventriculoarterial connections for CTA fetuses. The visualization rate for ventriculoarterial connections correlated with the volume quality. When the quality of volumes was classified as 1, only 18.5% CTA cases were successfully identified, whereas when the volume quality was between 2 and 5, the detection rate was between 92.9 and 100%. The distribution of the volumes quality was shown in Fig. 9. The mean score for volume quality during the second trimester was significantly higher than during late pregnancy (2.86 ± 1.09 vs. 1.40 ± 0.56; *P* < 0.01). The results suggested that the low detection rate for CTA fetuses by the 4D modality during the late pregnancy was caused by the low quality of 4D volumes acquired.

## Discussion

Perinatal care particularly in the case of ductus-dependent congestive heart diseases (CHDs) remains a challenge; therefore, early diagnosis of CTA is important to reduce morbidity and mortality [5, 21, 22]. According to the American Institute of Ultrasound in Medicine (AIUM), 4CV combined with two outflow tract views should be evaluated in routine prenatal screening examinations [23]. However, outflow tracts are not carefully examined in many screening programs. Many CTA fetuses that undergo routine prenatal ultrasound will have four symmetric chambers and the investigation always stops there; at most, two great arteries arising from two ventricles are visualized without ascertainment at to which of the arteries is the aorta versus the pulmonary artery. Furthermore, the examiner does not always visualize the full length of the outflow tract vessels. Therefore, it is challenging for a screening sonographer or an obstetric sonographer to obtain qualified cardiac images, especially for an inexperienced technician. In addition, interpretation must be done in real-time, either during the examination or after reviewing video clips, which also challenges most screening sonographers. The aforementioned challenges are underlying reasons for the low detection of CTA during routine screenings. Our study emphasized the necessity for identifying ventriculoarterial connections during prenatal screening and may provide some ideas to improve current screening methods.

STIC is not a new technique, but it has been shown to provide additional information for clinical evaluation of fetal cardiac anatomy [24]. Volume datasets are acquired from the fetal heart to enable appropriate post-processing. Images are acquired by a single automatic volume sweep, followed by the process of systematic analysis of image data according to their spatial and temporal domain. Dynamic image sequences are then extracted and displayed in a cine loop, which could be a multi-planar cross-sectional display and/or a surface-rendered display [16]. The examiner could then navigate within the volumes, re-slice, and get sufficient number of the standard planes for a comprehensive diagnosis [16].

Nowadays, 4D techniques are mainly used in fetal echocardiography but seldom used in prenatal screenings. We have used 4D STIC in fetal echocardiography since 2007 and the 4D technique has shown its value in helping diagnosis. We conducted this study with an aim to spur the use of 4D STIC in routine screenings. Uittenbogaard et al. [25] found that some key roles (i.e., fetal movement artifact, ROI setting, acquisition angle, fetal apex position, and fetal shadowing artifact) in volume acquisition may affect the volume quality, and thus proposed an acquisition condition scoring system. A study by Avnet et al. [26] demonstrated that trainees could get high quality volumes under the guidance of this condition scoring system. In fact, we have been performing volume acquisition in our obstetric screenings center since 2012. Screening sonographers were trained in a short-term session to familiarize with the acquisition conditions similar to that proposed by Uittenbogaard et al. [25] and no additional time out of the scheduled 30-min slots was needed to perform volume acquisition for fetus in the second trimester, either for normal or CHD fetuses. A training session including these acquisition conditions may obviously improve over time for the incorporation of STIC in routine screenings. In our experience, a clear 4CV on 2D is essential before commencing acquisition, and a clear image without distortion and shadows in Panel B confirms the quality of the volumes, which is reasonable when the acquisition angel is set to (GW + 5)°. Longer acquisition times with smaller angles assure maximal information in a 4D volume, while the acquisition time must decrease when fetal movement is frequent. Parameter settings should be made according to actual situation. However, volumes obtained in late pregnancy were not satisfactory as more shadows may present or useful information may not be included due to the large size of the fetus.

In fact, the great impediment for the application of 4D STIC in prenatal screening is volume process, which consumes much time and expertise. If the sonographers reformatting multi-planar volumes by rotating images in different panels with different angles do not have an effective algorithm, useful diagnostic views can still be retrieved but with much effort and difficulty. Our results showed an unsatisfactory detection rate and lower repeatability for 4D-1 modality, which suggested a limited value in routine screenings. Shih et al. [27] proposed a “Big-eyed frog” sign when they processed 4D volumes of fetal TGA and justified its value in improving diagnosis. However, the complex steps require higher experience and expertise and is better suitable for a specialist, and may be difficult to understand and manipulate by a screening sonographer.

In the current study, we proposed the protocol that could identify fetal ventriculoarterial connections by simply moving the reference point in three orthogonal planes without complex rotation of the images. When navigating the reference point, the great arteries were gradually revealed with its characteristics (i.e., the bifurcation of the pulmonary artery). For conventional 2D imaging, it is impossible for post-processing and only the original sonographic cuts were stored. If the characteristics of the great arteries are not well recorded, the screening sonographers are unable to make a correct diagnosis. It was also one of the reasons for the higher detection rate of 4D-2 modality in evaluating fetal TGA, when compared with the 2D method. In fact, screening sonographers can easily adapt to this protocol following a short few-days training session. This standardized method of processing 4D volumes is also more likely to be incorporated by beginners than acquiring many diagnostic views by the 2D method.

In the current study, we also included volumes of blood flow information to obtain the 4D–rendered images of the great arteries. The 4D–rendered images obtained from the 4D volumes obviously enhanced depth perception. These images reconstructed information and displayed a more complete and comprehensive picture. The data processing was very easy and the screening sonographers were apt to read the origin, course, and the spatial relationships of the great arteries. Therefore, inexperienced screening sonographers could be benefit from this technique given its ease. For cases of TGA, identification of the parallel great arteries when reviewing the 4D-rendered images was not challenging. Potential existing cardiac anomaly was then suspected. It is worth noting that the commitment of pulmonary arteries could be identified by the 4D–rendered images in some cases of TCA, while they could not be visualized by traditional 2D method. We must stress it is impossible to make a full diagnosis by just using a volume with blood flow information. Including a volume of gray-scale is necessary to evaluate cardiac anatomy in detail.

Usually, 4D STIC is not used alone in fetal echocardiography, but is considered a valuable method providing additional information during diagnosis. The echocardiographer or pediatrician reviews the 2D videos, and if needed, obtains the 4D images to help the diagnosis. In fact, accurate diagnosis cannot be made by just relying on a 4D volume without 2D images. Though standard cardiac views can be retrieved from volumes [28–31], the quality and resolution of these images were not as good as those from a 2D scan. Based on this point, we designed the combined method to test the feasibility of incorporating 4D volumes into routine screenings. After reviewing 2D images, the screening sonographers may have a preliminary judgement, thus could process the volumes more dedicatedly. At the same time, the sonographers may reevaluate the 2D images to determine whether there are some previously missed details after reviewing the 4D images. The results showed that the combined method could effectively improve the diagnostic accuracy in TGA, TOF, and TCA during the second trimester, when compared with using the 2D modality alone, which suggested the value for potential incorporation of the 4D STIC technique into routine screenings.

In the current study, our results suggested that 4D STIC did not help the diagnosis of fetal DORV. This may be due to the fact that DORV is a complex CHD with many classifications according to the spatial relationship of the great arteries. The ascending aorta and the pulmonary artery may lie side by side, or the aorta is on the right-anterior or left-anterior to the pulmonary artery, or the two great arteries are orthogonal to each other, similar to the situation of TOF. For the first three situations, the two great arteries were in parallel and were easy to distinguish from TOF. For the last situation, it was necessary to discern the sub-aortic conus. Our proposed protocol of volume analysis apparently could not provide an image of higher resolution than traditional 2D method and could not help the detection of the conus. Zidere et al. [32] published a report in which they demonstrated that the 4D–rendered images could provide more information and enhance prognostication with respect to the postnatal surgical approach. The authors made a detailed assessment of intracardiac anatomy, including the spatial relationship of the great arteries and the position of the VSD by post-processing the 4D STIC volumes. A pediatric cardiologist apparently could obtain more information from the 4D volumes than a screening sonographer.

The detection rate of the 4D modalities during late pregnancy was low. We evaluated the volume quality referenced in the scoring system by Goncalves et al. [20] and found that the mean score for the third trimester was significantly lower than the second trimester. The detection rate was likely correlated to volume quality, which was consistent with previous report [33]. As a retrospective study, we did not evaluate the acquisition quality as some factors (i.e., fetal movement) were not recorded. Furthermore, as the volumes used in the current study were acquired by an echocardiographer who was familiar with the acquisition conditions, it seemed that obtaining a valuable volume during late pregnancy was difficult, suggesting the limited value of 4D in late pregnancies.

It is important to stress that STIC has a few technical limitations. Volume quality apparently affects the diagnostic efficiency.Image distortion could be created by acoustic shadows, fetal movement artifact, and changes in heart rate. In our report, the poor quality of the 4D volumes in late pregnancy (29–36 GW) could have been caused by the increased acoustic shadows of the fetal ribs.

The study was limited in that we used the 4D volumes acquired by echocardiographers, not by screening sonographers. It might be impossible to observe large numbers of CHDs during routine screenings. As the largest fetal echocardiography center and consultation center in northeast China, we are able to obtain relatively large number of volumes of CTA fetuses. The current design, therefore, was intended to include more CHD fetuses to evaluate the utility of the proposed protocol in screening ventriculoarterial connections anomalies, which might compromise the study.

## Conclusion

In conclusion, we have proposed a step-by-step technique, the “tracing” technique, which allowed the examiner to identify fetal ventriculoarterial connections by 4D volumes. The inclusion of blood flow information into the volumes also improved the diagnosis. The addition of 4D STIC into routine screenings could improve the detection for TGA, TOF, and TCA. A prospective study in a large population is further warranted.

## Additional files

Additional file 1: **Video.** Multiplanar slicing of a fetus with normal cardiac structure of 19 gestational weeks. Panels A, B, and C represent three orthogonal planes (A, transverse; B, sagittal; and C, coronal). The cardiac apex in the 4CV was oriented upwards and the reference point was placed in the crux of the heart in Panel A. Move the reference point to the left ventricle in Panel A. By adjusting the position of the reference point in Panel A, near the position of the outflow tract (at the basal part of the left ventricle, near the crux of the heart), a great artery with clear course could be visualized in Panel B. Moving the reference point to this artery and then navigating along the course of the artery in Panel B, we could demonstrate a round (transverse) cross section of one great artery and a longitudinal section of the other great artery (characterized by the short trunk with bifurcation). They were aorta and the pulmonary artery. As the reference point was located at the aorta, it could be confirmed that aorta was originated from the left ventricle. (MOV 3179 kb)

Additional file 2: **Video.** Multiplanar slicing of a fetus with normal cardiac structure of 19 gestational weeks (the same fetus with Additional file 1). Panels A, B, and C represent three orthogonal planes (A, transverse; B, sagittal; and C, coronal). The cardiac apex in the 4CV was oriented upwards and the reference point was placed in the crux of the heart in Panel A. Move the reference point to the right ventricle in Panel A. By adjusting the position of the reference point in Panel A, near the position of the outflow tract (at the basal part of the right ventricle, near the crux of the heart), a great artery with its valve could be clearly visualized in Panel B. Moving the reference point to the valve in Panel B, two great arteries with cross section and longitudinal section respectively could be visualized in Panel A. As the reference point was located at the pulmonary artery, it could be confirmed that the pulmonary artery was originated from the right ventricle. (MOV 2846 kb)

Additional file 3: **Video.** Multiplanar slicing of TGA in a fetus of 25 gestational weeks. Panels A, B, and C represent three orthogonal planes (A, transverse; B, sagittal; and C, coronal). The cardiac apex in the 4CV was oriented upwards and the reference point was placed in the crux of the heart in Panel A. Move the reference point to the right ventricle in Panel A. By adjusting the position of the reference point in Panel A, a great artery connecting to the right ventricle could be visualized in Panel B. Moving the reference point to the valve and then navigating along the artery in Panel B, we could then confirm that aorta was originated from the right ventricle and the pulmonary artery (characterized by the short trunk with bifurcation) from the left in Panel A. The parallel relationship of the two great arteries was also demonstrated. (MOV 2560 kb)

Additional file 4: **Video.** Multiplanar slicing of DORV in a fetus of 26 gestational weeks. Panels A, B, and C represent three orthogonal planes (A, transverse; B, sagittal; and C, coronal). The cardiac apex in the 4CV was oriented upwards and the reference point was placed in the crux of the heart in Panel A. Move the reference point to the right ventricle in Panel A. By adjusting the position of the reference point in Panel A, a great artery connecting to the right ventricle could be visualized in Panel B. Moving the reference point to the valve and then navigating along the artery in Panel B, we could then confirm that aorta and the pulmonary artery (characterized by the short trunk with bifurcation) were both originated from the right ventricle and were coursing in parallel in Panel A. (MOV 3173 kb)

Additional file 5: **Video.** Multiplanar slicing of DORV in the fetus (the same with Additional file 4) using the “tracing” method in a different way. Panels A, B, and C represent three orthogonal planes (A, transverse; B, sagittal; and C, coronal). The cardiac apex in the 4CV was oriented upwards and the reference point was placed in the crux of the heart in Panel A. Moving the reference point to the right ventricle and navigating it to a certain point (close to the interventricularseptem, compared to the position mentioned in Additional file 4) in Panel A, a great artery connecting to the right ventricle could be visualized in Panel B. Moving the reference point to the valve and then navigating along the artery in Panel B, we could then confirm that aorta and the pulmonary artery (characterized by the short trunk with bifurcation) were both originated from the right ventricle and were coursing in parallel in Panel A. In this movie, the reference point was located at the pulmonary valve and the navigation was on the course of the main pulmonary artery. A subpulmoic ventricular septal defect could also be identified. (MOV 3818 kb)

Additional file 6: **Video.** Multiplanar slicing of TOF in a fetus of 24 gestational weeks. Panels A, B, and C represent three orthogonal planes (A, transverse; B, sagittal; and C, coronal). The cardiac apex in the 4CV was oriented upwards and the reference point was placed in the crux of the heart in Panel A. Move the reference point to the position of the outflow tracts (at the basal part of the ventricles, near the crux of the heart) in Panel A. By adjusting the position of the reference point in Panel A, a great artery could be visualized in Panel B. Moving the reference point to the valve and then navigating along the artery in Panel B, we could then confirm that aorta was originated from both left and right ventricles in Panel A. Navigating the reference point further along aorta in Panel B, we could visualize that the pulmonary artery (characterized by the short trunk with bifurcation) was originated from the right ventricle with a thickened valve in Panel A. (MOV 3452 kb)

Additional file 7: **Video.** Multiplanar slicing of TCA in a fetus of 28 gestational weeks. Panels A, B, and C represent three orthogonal planes (A, transverse; B, sagittal; and C, coronal). The cardiac apex in the 4CV was oriented upwards and the reference point was placed in the crux of the heart in Panel A. Move the reference point to the position of the outflow tracts (at the basal part of the ventricles, near the crux of the heart) in Panel A. By adjusting the position of the reference point in Panel A, a great artery could be visualized in Panel B. A VSD could also be identified. Moving the reference point to the valve and then navigating along the artery in Panel B, we could then confirm that only one great artery arising from both ventricles (mainly from the right ventricle) in Panel A. This great artery is aorta. The pulmonary artery was visualized arising from the root of aorta in Panel A. (MOV 2299 kb)
